# Integrated Neuroregenerative Techniques for Plasticity of the Injured Spinal Cord

**DOI:** 10.3390/biomedicines10102563

**Published:** 2022-10-13

**Authors:** Erik Leemhuis, Francesca Favieri, Giuseppe Forte, Mariella Pazzaglia

**Affiliations:** 1 Dipartimento di Psicologia, Sapienza Università di Roma, 00185 Rome, Italy; 2 Body and Action Lab, IRCCS Fondazione Santa Lucia, 00179 Rome, Italy; 3 Dipartimento di Psicologia Dinamica, Clinica e Salute, Sapienza Università di Roma, 00185 Roma, Italy

**Keywords:** spinal cord injury, neuroplasticity, spinal cord stimulation, neuroregeneration, stem cell therapy, nanomaterials, hydrogel, regenerative medicine

## Abstract

On the slow path to improving the life expectancy and quality of life of patients post spinal cord injury (SCI), recovery remains controversial. The potential role of the regenerative capacity of the nervous system has led to numerous attempts to stimulate the SCI to re-establish the interrupted sensorimotor loop and to understand its potential in the recovery process. Numerous resources are now available, from pharmacological to biomolecular approaches and from neuromodulation to sensorimotor rehabilitation interventions based on the use of various neural interfaces, exoskeletons, and virtual reality applications. The integration of existing resources seems to be a promising field of research, especially from the perspective of improving living conditions in the short to medium term. Goals such as reducing chronic forms of neuropathic pain, regaining control over certain physiological activities, and enhancing residual abilities are often more urgent than complete functional recovery. In this perspective article, we provide an overview of the latest interventions for the treatment of SCI through broad phases of injury rehabilitation. The underlying intention of this work is to introduce a spinal cord neuroplasticity-based multimodal approach to promote functional recovery and improve quality of life after SCI. Nonetheless, when used separately, biomolecular therapeutic approaches have been shown to have modest outcomes.

## 1. Introduction

Spinal cord injury (SCI) is a potentially highly disabling injury with exorbitant medical costs and is one of the most challenging diseases in the world to treat. It is difficult to evaluate how many people worldwide suffer from some type of SCI [1]. However, it is estimated that at least 250,000 new patients are diagnosed with SCI each year, and millions of people are currently living with the condition [2]. SCI can be classified as traumatic, caused by car accidents, falls, or violence, or nontraumatic, stemming from various causes, such as tumor-related compression, congenital disease, or spondylosis [3,4]. Depending on the severity of the injury, the main effect of SCI is the loss of motor and sensory functions in the parts of the body below the lesion [5]. In addition, there is a wide range of autonomic dysfunctions that unfold across different time periods, with effects on the respiratory, gastrointestinal, cardiovascular, immune, endocrine, and skeletal systems [6]. The presence of pain is a major problem after SCI, related to both the injury and stabilization measures and to changes in normal neurological activity [7]. Furthermore, SCI is a high risk factor for psychological disorders and depressive symptoms [8]. The enormous impact of an SCI on individuals and the entire community is evident.

Regardless of SCI severity, the lesion is permanent, and there is currently no cure. Acute therapeutic interventions are mostly limited to the stabilization of lesions and the prevention of further damage [9]. There is a strong commitment to finding a long-term solution that can restore damaged structures to their original integrity or full functionality. In any case, the primary goal of clinical intervention and intense research is to improve the quality of life (QOL) of these patients.

In this report, we attempt to address QOL improvement by offering an overview of the techniques and interventions that best contribute to the precise characterization of the neurological and functional status of an individual with SCI. Central to this narrative is the concept of neuroplasticity as a marker and founding process for adaptive neural reorganization that is compatible with the new physical conditions. However, this injury-induced spontaneous reorganization process can occur over a protracted time period and only partially contributes to functional recovery. By harnessing the reparative properties of biomolecular approaches, all possible types of interventions based on neural protection, regeneration, and modulation can be optimized to reduce over-reliance on medications that fail to impede or reverse the recovery process. The notion that the highly plastic spinal cord complements the plastic brain, both of which are connected to the activity of the physical body, brings to light an increasingly personalized therapeutic approach that can contribute to lasting change.

After providing some essential information on the pathophysiology of SCI, the first examples will illustrate new biomolecular approaches and reference the most recent experiences in nanotechnology, hydrogel, and stem cell transplantation therapy, which can ameliorate damage or regenerate neurological pathways between the brain and other parts of the body. Subsequent contributions come from research on neuromodulation and neural interfaces, which can re-establish efferent and afferent communications in interrupted sensorimotor loops. Finally, we highlight research evaluating the impact of behavioral, prosthetic, and robotic devices or physiotherapeutic procedures on neuroplasticity and address the importance of integrating several techniques. Combining various methodologies does not always produce the obvious effects of mutual enhancement and compensation of neuroplasticity and functional recovery processes. Therefore, in this work, we explore the pathophysiology, timing of functional recovery, and interactions between recent biomolecular advancements to spinal level and different types of interventions, both separately and in combination, that could offer promising therapeutic strategies for enhancing QOL after SCI.

## 2. Pathophysiology and Functional Recovery

### 2.1. Spinal Cord Injury Pathophysiology

SCI is a devastating trauma because it can disrupt the connection between the brain and peripheral organs, leading to a complex and highly heterogeneous pathophysiology [5,10,11]. The initial local damage is due to spinal cord compression, laceration, or contusion, often followed by hemorrhages and a cascade of events that lead to neuronal cell death [12]. Subsequently, numerous events, such as the formation of scar tissue and other molecular reactions, including local and global inflammatory reactions and demyelination, exacerbate the pathological condition and cause post-traumatic neural degeneration in the subsequent subacute phase, which ends with the chronic phase [13].

Notably, the severity of SCI depends on whether the lesion is complete or incomplete and, if incomplete, which part of the spinal cord is affected. The cervical tract of the spinal canal is smaller than the thoracic and lumbar regions. As such, even minor traumas can produce damage in the higher tract, resulting in acute complete sensorimotor tetraplegia (50% of cases). Lower-level traumatic SCIs are mostly caused by high energy traumas, resulting in higher rates of acute complete sensorimotor loss (70% of paraplegia cases) [14]. Most SCIs are incomplete, and even when complete, they may have spared spinal cord tissue that can be rescued and repaired to restore function. The main current treatment strategies include surgical interventions to anatomically stabilize and decompress the spinal cord, pharmacological and biological approaches to regenerate neurons through induction of tissue growth factors, and transplanting new cells, such as stem cells [15,16]. Treating SCI and improving functional recovery present significant obstacles due to the difficulty in delivering drugs across the blood–spinal cord barrier. In many cases, these medications meet low levels of compliance with rehabilitative measures and mostly affect vigilance, attention, and memory, contributing to the overall fatigue of patients [17].

In the acute phase, within the first three months, neurological recovery reaches the highest extent [18] while the highest functional improvements are delayed and observed within the first six months [19,20]. In the chronic phase post-injury, neurological recovery reaches a plateau, and sensorimotor deficits tend to remain permanent.

### 2.2. Functional Reorganization

Axotomy resulting from SCI has implications for the physiology of the cortical and corticospinal networks and may complicate or even impede the recovery process. Post-injury plasticity is not only associated with local neuronal and synaptic loss and inflammation from trauma, but also represents a change in activity-dependent plasticity resulting from the loss of regular activity in ascending and descending pathways. Resting-state functional magnetic resonance imaging studies have revealed that the strength of intra- and inter-hemispheric functional connectivity within sensorimotor networks increases or decreases in compensation for body sensory-motor deficits [21,22] and changes at different stages of injury [23]. This could potentially be a biomarker of functional recovery in SCI individuals [24]. Such reorganization is a dynamic process characterized by a combination of adaptive and maladaptive changes that influence recovery and clinical outcomes [25]. Therefore, optimizing functional recovery while minimizing maladaptive states after SCI is essential [26].

The recovery of motor control is a crucial element in properly reconnecting and adjusting to a physiological state to regain independence and reduce health complications resulting from prolonged inactivity [10,11,12,13,14]. The functional “silence” in the corresponding representative areas of the sensorimotor cortex seriously affects patients’ QOL and functional independence. Although significantly weaker, these brain representations of the deafferented body parts may be somatotopically preserved (finger somatotopy) [27]. Such preserved body representations have the potential to be used in functionally meaningful ways to acquire skilled neuroprosthetic control. Starting at the acute stage, a progressive macrostructural (atrophy) and microstructural degeneration of ascending or descending projections has also been observed [28], which may result in neurodegenerative processes in the subcortical and cortical structure. This not only directly impacts the sensorimotor system, but also other regions, such as the reduction of gray matter volume in the insular cortex [29] and hypoactivity of the anterior cingulate [30], which are influenced by visceral signals crucial for maintaining a homeostatic state via allostatic modulation. Nociceptive pain develops in 40–50% of patients and is associated with cortical and subcortical reorganization [31,32], and the amount of structural brain reorganization is correlated with the reported intensity of the pain [33]. Pain tends to become chronic.

However, a variety of pathways located in different parts of the white matter can allow some signals to reach the caudal spinal cord and represent a resource for long-term regeneration and functional recovery. Several attempts to treat the clinical symptoms of SCI have explored the possibility of redirecting neuronal activity in the corticospinal network. Finding ways to reconnect cortical motor neurons to their original targets remains a daunting, yet crucial, task. Activity-dependent plasticity may enhance the ability of denervated spinal circuits to respond to sparse descending signals and reorganize the topography and strength of their synaptic connections, which may also allow for more flexible signal redirection [34].

## 3. From Pharmacological to Stem Cell Interventions

### 3.1. Pharmacological and Nanotechnological Approach

**Figure 1:**
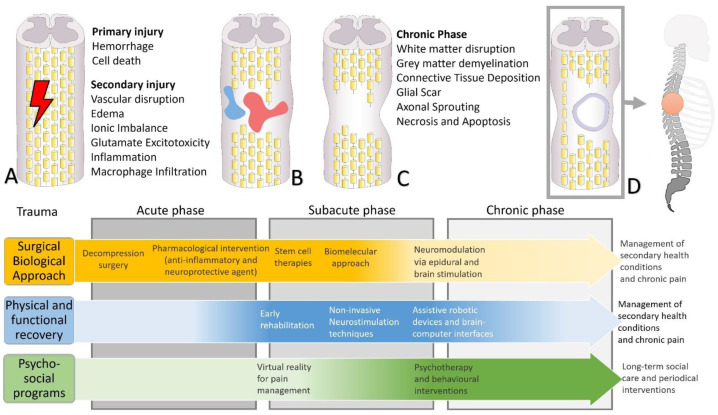
General overview of interventions for the treatment through the different phases of spinal cord injuries. (**A**) Traumatic event injures tissues, including axon bundles that run in the spinal cord, thus causing primary injury. (**B**) Acute phase: hemorrhage, oedema, and physiological inflammatory responses contribute to secondary injury, producing further damage to nerve tissue. Most surgical and pharmacological interventions are concentrated in this phase. The shock condition combined with the severe inflammatory condition negatively distorts the assessment of sensory and motor damage. (**C**) Sub-acute phase: reduction of pressure and inflammation due to surgical and pharmacological interventions allows better neurological assessment by more accurately assessing the level and severity of injury in terms of motor and sensory deficits. Possible early rehabilitation activities in limited cases. Possible psychological, training, and behavioral interventions. (**D**) Chronic phase: formation and stabilization of scar tissue; mild myelination, functional recovery, and improvement of symptoms possible. Training in the use of appropriate medical devices depends on the type of deficit acquired. Long-term important prevention or management of secondary medical conditions (cardiovascular, gastrointestinal, skeletal, neuropathic pain, etc.), and possible interventions to support psychological well-being and work/social reintegration.

At the pharmacological level, several approaches have been studied for their efficacy in neuroprotection and neuroregeneration to avoid nerve damage and promote regrowth or myelination of the nerves (Figure 1). In the SCI treatment regimen, neuroprotective agents prevent further nerve damage and reduce secondary damage; neurodegeneration agents promote neuronal regrowth or myelination [35]. The most commonly used neuroprotective drugs are methylprednisolone, naloxone, tirilazad, nimodipine, riluzole, minocycline, and fibro-blast growth factor, acting mainly on inflammatory processes and alteration in the vascular system due to the trauma [36]. Typical neurodegenerative medications are the granulocyte colony-stimulating factor, GM-1 ganglioside (Sygen), and cethrin, anti-Nogo, acting mainly to promote regeneration of injured neurons. Treatment using these drugs can provide minimal to moderate recovery of nerve function lost to SCI and reduce the risk of further loss of function [35]. Despite the large number of trials aimed to assess the efficacy of these medications on various SCI outcomes, there are some issues on the cost-effectiveness of the current pharmacologic treatment options that are related to collateral effects on secondary health outcomes and reduced efficacy on primary outcomes. The imbalanced microenvironment dynamically changes after SCI, acting mainly on a single mechanism and one target is not sufficient to treat the disease. It is possible that efficacy problems and adverse effects can, in part, not only be attributed to the substances themselves but also to the mode of use. For example, for better therapeutic efficacy, the timing of methylprednisolone administration in relation to the time elapsed since the traumatic event [36] and the modes of administration and transport to the site of the substance [37] seem to be of particular importance.

Advances in nanotechnology and nanomedicine have shown promising results in terms of improving pharmacological treatment.

In SCI, the nanotechnology approach represents a significant advancement [38], targeting neuroprotection and regeneration of tissues and networks impaired after SCI. The main benefit of nanotechnology is attributed to its ability to facilitate drug transport and overcome biological barriers to achieve its goals [9,38]. Preclinical studies provide promising results, especially when combination therapies target multiple mechanisms underlying SCI [9]. Several studies focused on specific combined substances, such as polymeric micelles, which appear to be effective in restoring lower-limb motor function and increasing neuronal survival, or carbon nanotubes, which appear useful for regenerative therapy and neuron repair. Substantially, drug-loaded nanoparticles rapidly infiltrate the injury site of the spinal cord in the acute phase, improving the regeneration microenvironment by reducing glial scars, neuronal death [39], and the overall neuroinflammation process [40,41]. Nanomaterials could act as therapeutic agents, releasing oxidative stress-related factors to treat the pain state after SCI and modifying the microenvironment to restore motor and sensory function. However, not all studies have reported the efficacy of nanotechnologies in SCI, indicating that other efforts and research are needed [42]. Therefore, it will be very interesting to monitor future developments in this field.

### 3.2. New Biomolecular Approaches

Recent research has bridged biomolecular science, engineering, and medical applications by focusing on the promising effect of hydrogel [43,44]. Hydrogel is a biomaterial characterized by a complex molecular network that allows it to absorb significant volumes of water (higher than 95%). This characteristic gives hydrogel molecules good mechanical proprieties, viscoelasticity, flexibility, high biocompatibility, and biodegradability, making them potentially useful in biomedical fields. As suggested by Ahmad et al. [43], various hydrogel molecules, including collagen, gelatin, and hyaluronic acid, have multiple uses and broad potentiality in fields such as wound dressing, controlled drug delivery, bone regeneration, tissue engineering, and biosensors. Moreover, the authors suggested that the use of hydrogel after SCI could have promising effects due to the biomaterial’s regeneration functions. Although it did not include a detailed analysis, this work demonstrated hydrogel’s potential to improve QOL after SCI, thanks to its possible advantages. As suggested by previous research, hydrogels may offer mechanical support for cells and tissues, promote cell migration and plasticity, and facilitate the long-term control of drug release [44]. All these aspects are associated with the long-term consequences of SCI and concern secondary health issues of SCI treatment, highlighting the importance of focusing on the application of these molecules in SCI. In fact, although the preliminary role of hydrogels has been considered regarding its effect on reducing scars, most recent studies have underlined its potentiality in preventing inflammatory responses and nerve compression typically associated with SCI after trauma [45,46]. The regeneration role of these molecules on nerves and neural circuits [47], especially in light of their association with stem cells and nanotechnologies [48,49], could lead to more effective SCI treatment with fewer side effects.

### 3.3. Stem Cells Approach

The utilization of various types of stem cells (SC) is one of the most promising candidates for regenerating injured tissues due to their capability to differentiate into several types of cell [50]. For this reason, they appear promising in spinal cord injury. Although these approaches are still being developed, studies on embryonic stem cells in animal models for SCI that have adopted in vitro manipulation and transplantation approaches have reported promising results in multiple neurological outcomes of trauma. SC of different origins, together with scaffolds, can release immunomodulating and neuroprotective factors which may support neuron survival, axonal growth, and control of glial scarring without significant side effects [16]. Evidence of nerve regeneration was reported in a preliminary study [51], indicating that stem cell treatment has the potential to help in SCI recovery.

In particular, the most promising effects are ascribed to adopting neural stem cells, which are highly effective in axon and neuronal repair due to their specific characteristics. Different types of cell therapy, such as induced pluripotent stem cells [52], mesenchymal stem cells [53], and neural stem cells [54], have been used in therapeutic SCI trials. All these stem cells are able to continue living in the host spinal cord and appear to contribute to nerve repair by differentiating into neuronal and glial cells [55,56,57].

There are also therapeutic applications of non-neural stem cells derived from bone marrow or adipose tissue, which are abundant in the body. Patients treated improved on ASIA sensory scores, including bladder function, with no serious adverse effects, demonstrating that it is a safe method for improving the QOL of patients with SCI.

Some evidence has reported neuronal repair following the transplantation of olfactory unsheathing cells [58,59] as viable candidates for improving neuropathic pain and motor function through multiple mechanisms that help axonal recovery, migration toward glial scars, and secretion of neurotrophic factors. However, their efficacy has been questioned due to the contradictory results reported [60,61].

Combining nanomaterials with SC is another innovative approach for therapeutic applications after SCI. Nanomaterial appears to be a promising platform for culturing the cells and SC-nanomaterial combination may hold further benefits for the patients, as suggested by a recent review of Zarepour et al. [62]. The authors analyzed the efficacy of nanomaterials in detail, both organic and inorganic, at multiple levels: From their carriers of therapeutic agents, anti-inflammatory and neurotrophic, but also in the form of scaffolds as in the field of hydrogels for tissue regeneration.

Despite extensive research exploring various cell-based therapies, including stem cells at different developmental stages in animal models, large clinical trials investigating the therapeutic efficacy of stem cell therapy in humans are lacking. These limits are ascribed to doubts about the safety–efficacy ratio of these types of therapies [15] and some related ethical concerns [50]. Additionally, there are concerns about the expense of developing adult stem cells, which seem to be the most effective in influencing regeneration after SCI. Another interesting area of research combining novel approaches is the application of nanomaterials, which could act as carriers for therapeutic agents or as platforms for culturing the cells [62].

Despite extensive research exploring different approaches to implement adoption of SC in SCI, some limitations are underlined. First, studies involving animal models are usually performed applying standardized protocols of lesions, treatments, and specific timing of transplantation in each group of investigators. These conditions are often unreplaceable in human patients with SCI. Therefore, completed human trials showed only limited results. On the one hand, SC seem to cause no harm and appear to be safe, showing no adverse reactions or side effects. Although studies on embryonic SC suggested possible immunosuppression side effect. On the other hand, results in terms of clinical outcomes were poor compared to expectations.

Clinical trials failed to keep their promising hypotheses and are still far from obtaining functional recovery and restoring neural circuits. Further studies are needed to improve our knowledge of their mechanisms of action. Combinatory strategies involving stem cells, biomaterials, and modifications of cell environment could be the key to translating fascinating premises into clinical practice.

## 4. From Neuromodulation to Sensorimotor Rehabilitation Interventions

### 4.1. Neuromodulation Approach

The neuromodulation consequent to electrical stimulation may inhibit or excite neural networks; it can also potentiate, sprout, and regenerate axonal neuronal interaction. Neuroplasticity-mediated functional recruitment represents the basis of modern neuromodulation techniques [63]. In patients with SCI, the complex system of ascending and descending circuits, and its residual signals, is the target of neuromodulation.

One of the approaches of neuromodulation adopted in SCI that exhibits the powerful neuroplasticity of the spinal cord is stimulation via deep (e.g., epidural) or transcutaneous approaches in different sections of the spinal cord. Clinical studies on spinal cord electrical stimulation have already shown different positive effects for these techniques [64,65,66].

Epidural spinal stimulation, which involves the surgical placement of electrodes onto the spinal cord’s dorsal surface [63], has been tested in patients with SCI to verify the effect on neuropathic pain. Evidence reported a reduction of pain symptoms in association with an improvement of motricity in the lower limb in patients with a level of impairment at the ASIA of A and B [35,64]. Studies reported some degree of benefit for spinal stimulation that also improves gastrointestinal mobility [67] and cardiac autonomic regulation [68], which are outcomes that can be detected further in SCI populations. Combined motor cortex and spinal cord stimulation is an example of leveraging different forms of spinal cord plasticity; structural, for the spinal axon terminals, and physiologic, for intrinsic spinal circuits. Two forms of electrical stimulation “top-down and bottom-up” to promote plasticity after SCI: neuromodulation of the motor cortex to increase the capacity of axon growth of neurons, and spinal stimulation to increase spinal neural activity. By augmenting sprouting, this approach helps mitigate the loss of descending cortical projections. Unlike the epidural method, transcutaneous stimulation is a non-invasive approach to spinal cord stimulation, which involves the placement of electrodes onto the surface of the patient’s skin. Furthermore, aside from this approach being less invasive, preliminary studies reported potential benefits in reducing muscle spasticity, improving residual motor functioning, and regulating autonomic functions, including heart rate, thermoregulation, and bladder sphincter function [69,70]. On top of experimental studies on animals, more clinical trials and human studies are needed to fully ascertain the advantages and long-term side effects of spinal cord stimulation for SCI [63].

Besides spinal cord stimulation techniques, brain stimulation is another approach to neuromodulation in SCI [63], which in some contexts, could represent a promising instrument for at least the maintenance and positive enhancement of post-trauma plasticity, if not for neurodegeneration. For example, non-invasive brain stimulation (NIBS), which has the advantages of being non-invasive and an easy operation to perform, and having broad clinical applications, represents a promising instrument for the regulation of the excitability of the cerebral cortex through electric or magnetic fields. Repetitive transcranial magnetic stimulation (rTMS) and transcranial direct current stimulation (tDCS) are two typical methods of NIBS, which can be adopted in SCI. Previous studies have shown the effect of NIBS in individuals with SCI, particularly on neuronal plasticity between the spinal cord and the brain [35]. Moreover, some studies highlight the availability of NIBS to treat neuropathic pain [71]. However, not all the studies have reported favorably for the effectiveness of the technique. Larger clinical trials and further research are needed to assess the applicability of these promising results.

### 4.2. Prosthetic and Neural Interfaces

Modern biomechanical approaches in SCI focus on the implementation of brain–computer interfaces (BCI). The principle of BCI is to create a new communication pathway between the brain and an effector without neuromuscular activation. In patients with SCI, brain–computer interface technology serves as an alternative or complementary user interface to close the sensorimotor loop. Examples of established human–machine interfaces depend on the user’s remaining abilities. In this way, efferent and afferent pathways are activated simultaneously, thereby stimulating the SCI area effectively. Through noninvasive EEG and invasive intracortical recordings, people can use brain activity to control BCI devices. Thus, patients can potentially activate electrical surface stimulators on the hand [72,73], walk [74], move a prosthetic or robotic limb [75,76], control bowel function [77], and restore the sense of touch [78], closing the sensorimotor loop [79]. These devices have improved prosthetic tool use, such as external robotic devices—Manipulators, wheelchairs, and exoskeletons—By combining mechanical properties with biostimulation through direct connection to paralyzed muscles [35,79]. Particularly, programs involving BCI are useful not only because they allow replacing functions (e.g., walking), but also due to their ability to generate beneficial neuroplastic effect [75,79,80]. Regaining the ability to move and to feel, in particular arms and hands, and using assistive devices is of paramount importance for people with spinal cord injuries, and for this reason BCI is an emerging strategy in rehabilitation programs [81]. The most pertinent example of personalization of an intervention is the use of ECG-based BCI to control an exoskeleton [82]. However, because this approach is highly invasive and extremely costly, it is not suitable for the needs of the larger population [83].

## 5. Constructing an Integrative Behavioral Approach for a Better Quality of Life

The concept of QOL for patients with SCI is elusive. Commonly, QOL refers to the subjective meaning of well-being and can take on a multitude of facets in the clinical setting (e.g., autonomy, self-efficacy, social life, and general well-being) [84]. Additionally, its implications are rooted in issues that are far more complex than any answer to a questionnaire can ever address. Among the commonly used measures of QOL [84] are the subjective satisfaction with life scale [85] and objective metrics for the physical, emotional, and social domains.

These objective measures, especially in cases of SCI, are closely related to typical complications, such as movement limitations, breathing difficulties, spasticity, musculoskeletal and neuropathic pain, and reduced or missing ability to control bladder, bowel, and sexual functions, all of which contribute to a profound reduction in the patient’s QOL [10]. Interestingly, among the 10 recommended scales of QOL for use in treating SCI of the SCIRE Project (http://www.scireproject.com/outcome-measures, accessed on 15 August 2022), SF-36 and LISAT are the most widely used [86].

While SF-36 is one of the most widely used measures of QOL in other patient populations, LISAT provides meaningful information on QOL for clinical and research purposes in the field of SCI. Advanced age, greater severity, and higher-level and lengthier duration of injury are associated with lower QOL [87,88,89]. From the perspective of clinicians, it is important to understand the priorities for functional recovery of individuals with SCIs in order that treatment and rehabilitation can be tailored to their needs [86]. Knowing the priorities of functional recovery expected for an improvement in QOL can provide a better understanding of the views and needs of SCI patients. Surveys on the priorities of functional recovery have been conducted in the USA and Europe [86]. Improving bladder and bowel function was ranked highly in the priorities of functional recovery. For patients with higher lesions, regaining arm and hand function was mentioned as the most important, while recovery of walking and sexual function was the highest priority for paraplegics. In a recent study on functional recovery priorities, most of the respondents were interested in trying advanced technology that would bring a significant improvement in their QOL [90]. Additionally, a better understanding of emotional and cognitive control could be critical for the selection of adaptive and flexible behavior [91]. What is becoming increasingly evident is how each event affects the whole organism and, consequently, the reorganization processes of its central nervous system. This is the basis for numerous research endeavors that focus on neuroplasticity as a direct or indirect clinical target in biological and functional recovery processes [92].

However, one piece still remains missing. As stated in the first section of this report, there is probably no event that does not come with some form of reorganization of the central nervous system. It is in the production of behavior, that is, in the interactions with the environment and the physical and social elements that constitute it, that the process of reciprocal body-brain regulation takes place in its most complete form.

In this perspective, physical activity certainly deserves to be mentioned first. Whether physical activity is practiced for strictly rehabilitative purposes, in informal settings, or for recreational purposes, it is perhaps the most striking example of how a behavior, especially one that is specific and repeated over time, is capable of becoming trophic and reorganizing activity in the nervous system. Rehabilitation is a foundational and indispensable element of almost every possible form of functional recovery in SCI [86,93]. As we will discuss next, many of the interventions presented need, or otherwise produce better results, when combined with appropriate forms of physical activity.

Simplifying the delivery of complex sensory stimuli through the latest technologies already makes it possible to envision highly individualized rehabilitation pathways. Virtual reality (VR) is probably one of the best examples, and it is possible to envisage its use throughout the rehabilitation path of SCI patients, both for standard and individualized procedures [94]. Preliminary results on the treatment of neuropathic pain are of particular interest [95]. Virtual walking sessions and limb movement imagery produced positive effects by reducing pain perception [96]. The virtual experience, when properly controlled, allows integration with other forms of sensory feedback, such as haptic and thermal, ensuring an immersive embodiment experience [97,98].

Mindfulness-based techniques basically consist of forms of meditation. In the clinical setting, they are proving to be a useful tool for controlling specific illnesses or symptoms, whether physical and psychological. An interesting aspect of such techniques is that they can have a significant impact on the functioning and structural organization of the central nervous system [99]. Mindfulness-based techniques are considered able to promote neural reorganization activity even following brief interventions [100]. In SCI patients, the presence of clinically relevant depressive conditions is high; additionally, anxiety-related disorders also benefited from mindfulness techniques [101].

VR is also shown to be useful in combination with stimulation techniques, such as transcranial direct current stimulation (tDCS). In a study with a pragmatic approach, the cost-benefit ratio of tDCS and VR interventions taken individually or in combination compared with standard care is evaluated [102]. After three months, at the end of the interventions, tDCS+VR patients report lower neuropathic pain and better QOL with only a slightly higher cost than the other conditions. One year later, however, patients in the tDCS+VR group in addition to having a better overall condition cost less overall than the other groups indicating less access to health system resources.

Another approach that aims to improve the QOL is to provide technologically advanced tools that can deliver an enhanced autonomy and social life [103,104]. An emblematic and particularly topical case is that of powered lower-limb exoskeletons (EXOs). Although in home and social settings, EXOs still have significant limitations, they are increasingly being adopted for rehabilitation purposes in the clinical setting. In participants with chronic SCI, prolonged use of EXOs has improved the QOL attributable primarily to decreased neuropathic pain and improved bladder management [105]. However, other experiences have found discordant results that do not recognize long-term pain reduction [40]. Instead, the same study identified improvements in balance and muscle spasticity that are likely probably dependent on neuroplasticity promoted by the activities performed and related to the level and severity of injury.

## 6. Therapeutic Strategies and Innovative Biotechnological Opportunities

Given the mechanisms underlying neuroplastic processes, a systematic integration of interventions promoting neuronal plastic activity and rehabilitation would be optimal [106]. In such a scenario, animal models are particularly useful in providing insights into the potential improvements produced by certain procedures. For example, treatments based on the chondroitinase ABC enzyme or anti-Nogo-A were found to be most effective when used in combination [107]. However, when used in synergy with physiotherapy, significant differences were identified at the cellular level (i.e., sprouting and axon regeneration) and in terms of functional improvement in the SCI models used. Zhao et al. also showed that, when independently combined with motor rehabilitation, the two compounds produced similar functional improvement but used different mechanisms to promote axon regeneration [107].

Garcia-Alias et al. [108] previously identified the positive impact of the combination of the chondroitinase ABC enzyme and motor rehabilitation; what is of particular interest is the consideration that the positive effect appeared to be a task-specific one. According to the authors, the administration of the treatment opened a “window” of potential reorganization that could be exploited through specific activities. However, “one behavior training makes another behavior worse” [108].

During rehabilitation, only the motor functions that were addressed benefited; those that were excluded were at risk of depotentiation, showing competition between various patterns of newly formed neural circuitry for a specific functional control.

Starting from a similar conceptual basis, Nagappan et al. [109] used concepts of “brake” and “facilitator” to indicate all processes that aimed to regulate neuroplastic activity to ensure stability or increase neuronal adaptive capacity under healthy conditions. The authors pointed out how such processes may differ depending on whether they are related to the central nervous system or the peripheral nervous system. Particularly relevant is their proposal of an approach aimed at modulating neuroplastic stability to obtain the fullest synergy in specific pharmacological, neural stimulation, and physiotherapeutic therapies.

The attractive biomolecular approaches to SCI therapy have not yet had an impact on clinical outcomes. For SCI treatment, a number of aspects must be taken into account, including the treatment time window, the severity of the injury, individual differences, and other factors. After injury, the initial period aims to ensure that the patient receives the best possible care, starting with neurological stabilization, to avoid secondary complications (see Figure 1 for a general overview of interventions). The next stage should consider key biomolecular findings on neural tissue regeneration from randomized controlled trials that have a highly favorable biosafety profile and offer a potential program to ensure predictable improvement in motor and sensory performance. Based on this knowledge, studies that explore the complex pathological mechanisms of SCI and combine biological tissue engineering or cell transplantation strategies, with simultaneous multitarget, multimodal, multistage interventions may provide a good framework for treating SCI. In recent decades, the number of findings has increased exponentially without a corresponding evolution in the treatments offered to patients. It is time to integrate multiple interventions to maximize outcomes for individual patient needs and translate research findings into the practice of SCI neurorehabilitation.

## 7. Conclusions

What is presented in this paper does not purport to be exhaustive in identifying the tools that are available for the treatment of SCI, as well as the many targets on which they act. Although considerable progress has been made throughout the long clinical journey, in terms of pharmacological interventions, neuromodulation through stimulation techniques, or technologically advanced aids or physiotherapy, the impact on patients’ QOL remains limited. The integration between techniques seems to be an interesting avenue for further evaluation in both research and clinical set-ups for the achievement of a high QOL and for understanding the biological mechanisms underlying neuroplasticity.
